# Risk factors for nosocomial SARS-CoV-2 infections in patients: results from a retrospective matched case–control study in a tertiary care university center

**DOI:** 10.1186/s13756-022-01056-4

**Published:** 2022-01-17

**Authors:** Seven Johannes Sam Aghdassi, Frank Schwab, Luis Alberto Peña Diaz, Annika Brodzinski, Giovanni-Battista Fucini, Sonja Hansen, Britta Kohlmorgen, Brar Piening, Beate Schlosser, Sandra Schneider, Beate Weikert, Miriam Wiese-Posselt, Sebastian Wolff, Michael Behnke, Petra Gastmeier, Christine Geffers

**Affiliations:** 1 Charité-Universitätsmedizin Berlin, corporate member of Freie Universität Berlin and Humboldt Universität zu Berlin, Institute of Hygiene and Environmental Medicine, Hindenburgdamm 27, 12203 Berlin, Germany; 2 Berlin Institute of Health at Charité – Universitätsmedizin Berlin, BIH Biomedical Innovation Academy, BIH Charité Digital Clinician Scientist Program, Anna-Louisa-Karsch-Straße 2, 10178 Berlin, Germany

**Keywords:** SARS-CoV-2, Healthcare-associated infection, Case–control study, Contact tracing, Infection control, Hospital epidemiology

## Abstract

**Background:**

Factors contributing to the spread of SARS-CoV-2 outside the acute care hospital setting have been described in detail. However, data concerning risk factors for nosocomial SARS-CoV-2 infections in hospitalized patients remain scarce. To close this research gap and inform targeted measures for the prevention of nosocomial SARS-CoV-2 infections, we analyzed nosocomial SARS-CoV-2 cases in our hospital during a defined time period.

**Methods:**

Data on nosocomial SARS-CoV-2 infections in hospitalized patients that occurred between May 2020 and January 2021 at Charité university hospital in Berlin, Germany, were retrospectively gathered. A SARS-CoV-2 infection was considered nosocomial if the patient was admitted with a negative SARS-CoV-2 reverse transcription polymerase chain reaction test and subsequently tested positive on day five or later. As the incubation period of SARS-CoV-2 can be longer than five days, we defined a subgroup of “definite” nosocomial SARS-CoV-2 cases, with a negative test on admission and a positive test after day 10, for which we conducted a matched case–control study with a one to one ratio of cases and controls. We employed a multivariable logistic regression model to identify factors significantly increasing the likelihood of nosocomial SARS-CoV-2 infections.

**Results:**

A total of 170 patients with a nosocomial SARS-CoV-2 infection were identified. The majority of nosocomial SARS-CoV-2 patients (n = 157, 92%) had been treated at wards that reported an outbreak of nosocomial SARS-CoV-2 cases during their stay or up to 14 days later. For 76 patients with definite nosocomial SARS-CoV-2 infections, controls for the case–control study were matched. For this subgroup, the multivariable logistic regression analysis revealed documented contact to SARS-CoV-2 cases (odds ratio: 23.4 (95% confidence interval: 4.6–117.7)) and presence at a ward that experienced a SARS-CoV-2 outbreak (odds ratio: 15.9 (95% confidence interval: 2.5–100.8)) to be the principal risk factors for nosocomial SARS-CoV-2 infection.

**Conclusions:**

With known contact to SARS-CoV-2 cases and outbreak association revealed as the primary risk factors, our findings confirm known causes of SARS-CoV-2 infections and demonstrate that these also apply to the acute care hospital setting. This underscores the importance of rapidly identifying exposed patients and taking adequate preventive measures.

## Background

Healthcare-associated infections (HAIs) represent one of the frequently occurring adverse events in the practice of medicine [1–3], with lower respiratory tract infections (LRTIs) being among the most prevalent HAIs and entailing a significant burden of disease [4–6]. Bacterial pathogens are the most commonly recorded causing microorganisms of nosocomial LRTIs [7, 8]. However, particularly during respiratory virus seasons, nosocomial LTRI outbreaks with viral pathogens occur regularly [9, 10]. To provide healthcare facilities with useful mitigation strategies for respiratory viruses, the clinical practice guidelines for seasonal influenza were published by the Infectious Diseases Society of America [11]. Nevertheless, infection prevention and control (IPC) and surveillance activities in the past have predominantly focused on bacterial HAIs, for instance by promoting hand hygiene campaigns in multiple countries [12, 13]. Accordingly, evidence generated concerning risk factors for nosocomial LRTIs and recommendations on how to prevent them, has been largely focused on bacterial infections. In the context of the COVID-19 pandemic, this perspective has shifted towards viral transmission.

Despite a large amount of data demonstrating a variety of risk factors for SARS-CoV-2 infections in the general population [14–17], and multiple reports of SARS-CoV-2 outbreak in healthcare facilities [18–22], data concerning risk factors for nosocomial SARS-CoV-2 infections in hospitalized patients remain limited. Given that hospitalized patients represent a particularly vulnerable group and that nosocomial SARS-CoV-2 cases and especially clusters of nosocomial cases often lead to significant disruptions in health care provision [23–25], it is essential to better understand factors that increase the risk of nosocomial SARS-CoV-2 infections.

Consequently, the study at hand had two principal objectives. First, we aimed to retrospectively identify all potentially nosocomial SARS-CoV-2 cases that occurred in a defined time period, and describe their characteristics. Second, by means of a matched case–control study, we sought to determine risk factors for nosocomial SARS-CoV-2 infections in hospitalized patients. Insights generated from these analyses may inform targeted approaches to the prevention of nosocomial SARS-CoV-2 infections.

## Methods

### Setting

The study was based on retrospective data of patients admitted to Charité university hospital during a nine-month period (May 1, 2020 – January 31, 2021). Charité is a tertiary care university center with three separate sites and over 3,000 patient beds. During the study period, a hospital-wide policy was in place, requiring all patients to be screened for SARS-CoV-2 via reverse transcriptase polymerase chain reaction (RT-PCR) from a combined oro- and nasopharyngeal swab on the day of admission, or shortly before in case of elective admissions. No policy was in place to repeat tests at regular intervals, but additional testing was conducted when deemed necessary, for instance in reaction to symptoms developing, or in the context of contact tracing and outbreak management. In response to the pandemic, enhanced standard precautions were put in place at Charité in March 2020 by requesting all staff to wear a medical face mask at all times when interacting with patients or colleagues. The local IPC team investigated all SARS-CoV-2 cases. Potentially nosocomial cases were documented in detail and usually prompted extensive testing of all potentially exposed patients and staff, as well as thorough contact tracing, both retrospective (i.e. in search of a probable source) and prospective (i.e. to identify persons exposed). All data acquisition and subsequent analyses were performed in alignment with the German Protection Against Infection Act that requires all hospitals in Germany to collect and analyze data on healthcare-associated infections [26]. Therefore, ethical approval and informed consent were not required.

### Inclusion criteria for nosocomial SARS-CoV-2 cases

All patients admitted to Charité during the study period were included in the study. Patients that fulfilled the following requirements were defined as nosocomial SARS-CoV-2 cases: a negative SARS-CoV-2 RT-PCR test on the day of admission or up to two days prior to admission, no suspected SARS-CoV-2 infection at the time of admission, and a positive SARS-CoV-2 RT-PCR test on day five or later after admission. The day of admission was counted as day one. Accordingly, laboratory results between April 29, 2020 (i.e. two days before the first day of the study period), and February 13, 2021 (i.e. 14 days after the last day of study period), were considered. To acquire the necessary virology results, the data warehouse (“Hygieneportal”), maintained by the informatics team of Charité’s Institute of Hygiene and Environmental Medicine and routinely utilized for the automated cluster alert system “CLAR” [27], was employed.

### Subgroup of definite nosocomial cases and case–control study

For the purpose of this study, cases fulfilling the above-stated criteria, and where the first positive SARS-CoV-2 test was from a sample taken more than 10 days after hospital admission, were considered “definite” nosocomial cases. The decision for this cut-off was made based on available literature concerning the length of the incubation period [28, 29]. For this subgroup, a matched case–control study with a one to one ratio of cases and controls was conducted. A set of matching criteria were applied when sampling the controls:

1. First, a control must not have a positive SARS-CoV-2 RT-PCR test during the study period (criterion “negativity”).
2. Second, a control had to be hospitalized for at least the same number of days that had passed between admission and first positive SARS-CoV-2 test of the associated case. For cases that tested positive more than 14 days after admission, controls had to be hospitalized for a minimum of 14 days (criterion “length of hospital stay”).
3. Third, a control had to be admitted to the same in-patient ward (excluding emergency departments) as the case, within seven days after or before the admission of the case. If a patient was admitted via an emergency department, the first non-emergency (i.e. in-patient) ward was regarded as the admission ward and utilized for the sampling process (criterion “admission ward”).

Where more than one control was identified, further selection criteria were applied. Controls admitted after the associated case were chosen over those admitted before. Where more than one suitable control remained after applying all above-stated steps, the control with an admission date closest to that of the associated case was selected. Cases without matched controls were excluded from the case–control study. Due to the exploratory nature of the study, a sample size was not calculated.

For every SARS-CoV-2 case and control, a relevant timeframe was defined, for which information was collected. For cases, this was the day of the positive test minus 14 days. For controls of cases that tested positive more than 14 days after admission, it was the day of admission plus 14 days. For controls of cases that tested positive less than 14 days after admission (on “day X”), the relevant timeframe spanned from “day of admission minus (14-X)” to “day of admission plus X”. These specifications were made to ensure that the relevant timeframe for all cases and controls was 14 days, and that the number of hospitalization days during the relevant timeframe was also identical between cases and respective controls.

### Recorded parameters

For all nosocomial SARS-CoV-2 patients and all sampled controls, the following parameters were collected:

- Age at the beginning of the relevant timeframe
- Sex
- Charlson Comorbidity Index
- Admission via emergency department (yes/no)
- Ward specialty of first in-patient ward (excluding emergency departments)
- Intensive care unit stay during the relevant timeframe (yes/no)
- Number of documented procedure codes in the patient files during the relevant timeframe
- Number of ward transfers during the relevant timeframe
- Number of patient room transfers during the relevant timeframe
- Number of contact patients (defined as patients placed in the same patient room for any duration) during the relevant timeframe
- Number of contact patient-days (product of contact patients and the duration of contact in days) during the relevant timeframe
- Presence at a ward with a SARS-CoV-2 outbreak reported to authorities during the stay or up to 14 days later (yes/no)
- Documented contact to a (suspected) SARS-CoV-2 case during the relevant timeframe

Information on patient movements, including transfers, were extracted from the “Hygieneportal” system. Patients with known contact to SARS-CoV-2 cases were systematically recorded by the IPC team throughout the study period. This information was either saved locally (e.g. in Excel tables) or in the “Hygieneportal” database. Outbreaks were reported to health authorities at the discretion of the hospital IPC team. Usually, if two or more SARS-CoV-2 infections occurred that appeared to be epidemiologically linked, this was considered an outbreak and a report to the responsible public health office was made.

For nosocomial SARS-CoV-2 patients, the following additional parameters were included:

- Difference in days between hospital admission and first positive test
- Ward specialty of ward with first positive test
- Assessment of the presumed source of infection by the IPC physician in charge

### Analytic approach

Data for all nosocomial cases were collected and described. Definite nosocomial cases with matched controls and their respective controls were further analyzed in a case–control study. Descriptive analyses examined differences in clinical and epidemiological factors between cases and controls. Paired Wilcoxon signed-rank test was used to compare continuous variables and chi-squared test was utilized for categorical variables. The primary analysis examined potential risk factors for nosocomial SARS-CoV-2 acquisition. We calculated crude and adjusted odds ratios (OR) with 95% confidence intervals (CI95) (Wald test) for the outcome nosocomial SARS-CoV-2 acquisition employing logistic regression models. Differences in recorded parameters between cases and controls were analyzed by a multivariable regression analysis using conditional logistic regression with a stepwise variable selection. Parameters were included in the model by stepwise forward variable selection with a significance level of p = 0.05 for including a parameter in the model and p = 0.1 for a parameter to remain in the model.

All analyses were exploratory and performed with IBM SPSS (version 25) and SAS (version 9.4) [SAS Institute, Cary, NC, USA].

## Results

**Fig. 1:**
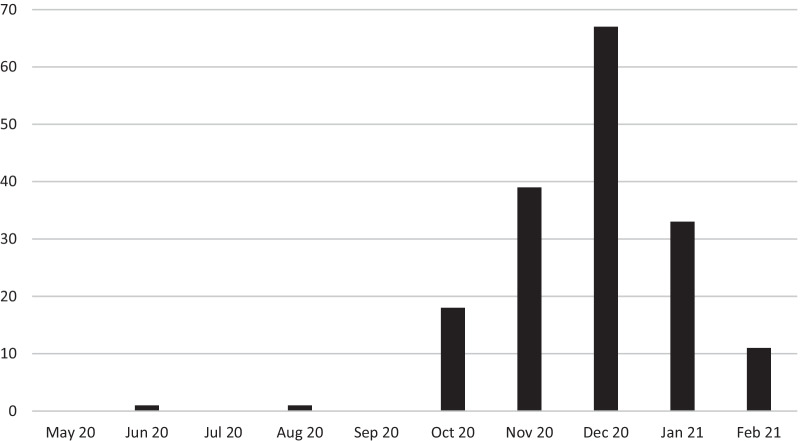
Number of nosocomial SARS-CoV-2 cases per month in patients treated between May 2020 and January 2021. Annotations: Patients admitted to the hospital until January 31, 2021 were included in the study. As the graph pertains to the date of the first positive test, values of greater than zero for February 2021 are possible

Between May 2020 and January 2021, 128,758 patients were admitted to Charité and 170 nosocomial SARS-CoV-2 cases in patients were recorded. This corresponds to an incidence of 0.1 per 100 admissions. During the study period, 2,192 in-patients with a positive SARS-CoV-2 RT-PCR result or coded coronavirus disease (ICD-10 code U07.1) were treated at Charité, of which nosocomial cases (n = 170) accounted for approximately 8%. The majority of nosocomial SARS-CoV-2 cases occurred during the months November 2020, December 2020 and January 2021 (n = 139, 82%). Figure 1 depicts the number of nosocomial SARS-CoV-2 cases aggregated per month over the course of the study period.

The median time between hospital admission and positive SARS-CoV-2 test for all nosocomial cases was 11 days (interquartile range 7–17). The majority of nosocomial SARS-CoV-2 patients had been treated at wards that reported an outbreak of nosocomial SARS-CoV-2 cases during their stay or up to 14 days later (n = 157, 92%). Despite extensive investigation, no contact to confirmed or suspected SARS-CoV-2 cases was documented for 77 nosocomial SARS-CoV-2 patients (45%). Conversely, in 93 cases (55%), contact to SARS-CoV-2 infected individuals was documented. Only in one case contact occurred prior to hospital admission, in all other (n = 92) cases contact took place during the hospitalization. Further characteristics of the 170 nosocomial SARS-CoV-2 cases can be found in Table 1. For 124 patients (73%), the IPC team was able to determine the probable source of infection, with presence of SARS-CoV-2 positive patients or staff at the ward being the most frequently assumed cause of infection (n = 117 of 124, 94%). In seven cases, individuals not associated to the ward (e.g. visitors) were seen as the likely source of infection.

**Table 1:** Characteristics of all nosocomial SARS-CoV-2 cases in patients treated between May 2020 and January 2021 (n = 170)

Parameter	Category (where applicable)	Number (%) or median (IQR)
Difference in days between hospital admission and first positive test		11 (7–17)
Age	Years	70 (58–80)
Sex	Female	78 (45.9%)
	Male	92 (54.1%)
Charlson Comorbidity Index		6 (4–8)
Admission via ER	Yes	101 (59.4%)
Ward specialty of first in-patient ward (excluding ER)	MED	66 (38.8%)
	SUR	40 (23.5%)
	HEM	20 (11.8%)
	PSY	9 (5.3%)
	OTH	9 (5.3%)
	ICU	26 (15.3%)
Ward specialty of ward with first positive test	MED	76 (44.7%)
	SUR	53 (31.2%)
	HEM	19 (11.2%)
	PSY	9 (5.3%)
	OTH	5 (2.9%)
	ICU	8 (4.7%)
ICU-stay during the relevant timeframe	Yes	43 (25.3%)
Number of documented procedure codes in the patient files during the relevant timeframe		6 (3–10)
Number of ward transfers during the relevant timeframe		1 (0–2)
Number of patient room transfers during the relevant timeframe		2 (1–3)
Number of contact patients during relevant timeframe		4 (2–7)
Number of contact patient-days during relevant timeframe		17 (9–28)
Present at a ward with a SARS-CoV-2 outbreak reported to authorities during the stay or up to 14 days later	Yes	157 (92.4%)
Documented contact to a (suspected) SARS-CoV-2 case during the relevant timeframe	Yes	93 (54.7%)
Documented contact to a (suspected) SARS-CoV-2 case during the relevant timeframe before hospital admission	Yes	1 (0.6%)
Documented contact to a (suspected) SARS-CoV-2 case during the relevant timeframe after hospital admission	Yes	92 (54.1%)
Documented contact to (suspected) SARS-CoV-2 positive patients during the relevant timeframe*	Yes	68 (40%)
Documented contact to (suspected) SARS-CoV-2 positive staff during the relevant timeframe*	Yes	32 (18.8%)

For SARS-CoV-2 cases, the relevant timeframe was defined as the 14 days preceding the first positive test. Age represents the age in years at the beginning of the relevant timeframe. Contact patients were defined as patients placed in the same patient room for any period of time. Contact patient-days are the product of contact patients and the duration of contact in days. Abbreviations: ER – emergency room; ICU – intensive care unit; IQR – interquartile range; HEM – Hematology/Oncology (non-ICU); MED – internal medicine (non-ICU) (incl. dermatology, geriatrics, neurology); OTH – other/mixed (non-ICU); PSY – psychiatry/psychosomatry; SUR – surgery (non-ICU) (incl. gynecology, urology). *Contact to more than one group of SARS-CoV-2 positive persons (e.g. patients and staff) was possible. In that case, both were recorded and counted

For 76 patients with considered definite nosocomial SARS-CoV-2 infection, controls were matched for the case–control study. The descriptive analysis of the case–control study revealed no significant differences regarding patient demographics and underlying characteristics (Table 2). The univariable analysis demonstrated that presence at a ward that experienced a SARS-CoV-2 outbreak (OR: 14.5 (CI95: 3.5–60.8)), and documented contact to SARS-CoV-2 cases (OR: 21.5 (CI95: 5.2–88.8) were significantly associated with nosocomial SARS-CoV-2 infection, with documented contact to infected patients showing the strongest association (OR: 28 (CI95: 3.8–205.8) (Table 3). The multivariable logistic regression analysis for the outcome nosocomial SARS-CoV-2 acquisition confirmed the results of the univariable analysis by revealing presence at a ward that experienced a SARS-CoV-2 outbreak (OR: 15.9 (CI95: 2.5–100.8)) and documented contact to SARS-CoV-2 cases (OR: 23.4 (CI95: 4.6–117.7)) to be the principal risk factors for nosocomial SARS-CoV-2 infections in patients (Table 4).

**Table 2:** Descriptive analysis of definite nosocomial SARS-CoV-2 cases with matched controls (n = 76) and their respective controls (n = 76) for the outcome nosocomial SARS-CoV-2 acquisition

Parameter	Category (where applicable)	Number (%) or Median (IQR)		*p *value
		Definite SARS-CoV-2 case	Control	
Age	Years	70 (61–82)	70 (58–78)	0.362
Sex	Female	36 (47.4%)	36 (47.4%)	1.000
	Male	40 (52.6%)	40 (52.6%)	
Charlson Comorbidity Index		6 (5–8.5)	6 (4–9)	0.647
Admission via ER	Yes	44 (57.9%)	44 (57.9%)	1.000
	No	32 (42.1%)	32 (42.1%)	
Ward specialty of first in-patient ward (ER)	SUR	14 (18.4%)	14 (18.4%)	1.000
	MED	25 (32.9%)	25 (32.9%)	
	ICU	18 (23.7%)	18 (23.7%)	
	HEM	9 (11.8%)	9 (11.8%)	
	PSY	5 (6.6%)	5 (6.6%)	
	OTH	5 (6.6%)	5 (6.6%)	
Ward specialty of ward with first positive test	SUR	24 (31.6%)		
	MED	33 (43.4%)		
	ICU	4 (5.3%)		
	HEM	9 (11.8%)		
	PSY	5 (6.6%)		
	OTH	1 (1.3%)		
ICU-stay during the relevant timeframe	Yes	26 (34.2%)	29 (38.2%)	0.613
Number of documented procedure codes in the patient files during the relevant timeframe		9 (5–14)	12 (9–17)	< 0.001
Number of ward transfers during the relevant timeframe		1 (0–2)	1 (1–2)	0.892
Number of patient room transfers during the relevant timeframe		2 (1–3)	2 (1–3)	0.449
Number of contact patients during relevant timeframe		5 (2.5–8)	5.5 (3–8.5)	0.343
Number of contact patient-days during relevant timeframe		22 (12–36)	30 (19–39.5)	0.055
Present at a ward with a SARS-CoV-2 outbreak reported to authorities during the stay or up to 14 days later	Yes	70 (92.1%)	43 (56.6%)	< 0.001
Documented contact to a (suspected) SARS-CoV-2 case during the relevant timeframe	Yes	45 (59.2%)	4 (5.3%)	< 0.001
Documented contact to a (suspected) SARS-CoV-2 case during the relevant timeframe before hospital admission	Yes	0 (0%)	0 (0%)	n.d
Documented contact to a (suspected) SARS-CoV-2 case during the relevant timeframe after hospital admission	Yes	45 (59.2%)	4 (5.3%)	< 0.001
Documented contact to (suspected) SARS-CoV-2 positive patients during the relevant timeframe*	Yes	30 (39.5%)	3 (3.9%)	< 0.001
Documented contact to (suspected) SARS-CoV-2 positive staff during the relevant timeframe*	Yes	16 (21.1%)	1 (1.3%)	< 0.001

Nosocomial SARS-CoV-2 cases, where the first positive SARS-CoV-2 test was from a sample taken more than 10 days after hospital admission, were considered definite nosocomial cases. For SARS-CoV-2 cases, the relevant timeframe was defined as the 14 days preceding the first positive test. For controls, the relevant timeframe comprised of 14 days. Age represents the age in years at the beginning of the relevant timeframe. Contact patients were defined as patients placed in the same patient room for any period of time. Contact patient-days are the product of contact patients and the duration of contact in days. Abbreviations: ER – emergency room; ICU – intensive care unit; IQR – interquartile range; HEM – Hematology/Oncology (non-ICU); MED – internal medicine (non-ICU) (incl. dermatology, geriatrics, neurology); n.d. – not defined; OTH – other/mixed (non-ICU); PSY – psychiatry/psychosomatry; SUR – surgery (non-ICU) (incl. gynecology, urology). *Contact to more than one group of SARS-CoV-2 positive persons (e.g. patients and staff) was possible. In that case, both were recorded and counted

**Table 3:** Univariable logistic regression analysis of definite nosocomial SARS-CoV-2 cases with matched controls (n = 76) and their respective controls (n = 76) for the outcome nosocomial SARS-CoV-2 acquisition

Parameter	Characteristic	Odds ratio (CI95)	*p *value
Age	per year	1.01 (0.99–1.04)	0.301
Sex (male vs. female)	male	1 (0.52–1.92)	1.00
Charlson Comorbidity Index	per score point	1.03 (0.93–1.14)	0.573
Admission via ER (yes vs. no)	yes	1 (0.45–2.23)	1.00
ICU-stay during the relevant timeframe (yes vs. no)	yes	0.73 (0.29–1.81)	0.493
Number of documented procedure codes in the patient files during the relevant timeframe	per additional procedure code	0.94 (0.9–0.99)	0.017
Number of ward transfers during the relevant timeframe	per additional ward transfer	1.02 (0.76–1.38)	0.880
Number of patient room transfers during the relevant timeframe	per additional room transfer	0.93 (0.76–1.14)	0.509
Number of contact patients during relevant timeframe	per additional contact patient	0.97 (0.88–1.06)	0.439
Number of contact patient-days during relevant timeframe	per additional contact patient-day	0.99 (0.97–1.01)	0.147
Present at a ward with a SARS-CoV-2 outbreak reported to authorities during the stay or up to 14 days later (yes vs. no)	yes	14.5 (3.46–60.77)	< 0.001
Documented contact to a (suspected) SARS-CoV-2 case during the relevant timeframe (yes vs. no)	yes	21.5 (5.21–88.75)	< 0.001
Documented contact to a (suspected) SARS-CoV-2 case during the relevant timeframe before hospital admission (yes vs. no)	yes	n.d	
Documented contact to a (suspected) SARS-CoV-2 case during the relevant timeframe after hospital admission (yes vs. no)	yes	21.5 (5.21–88.75)	< 0.001
Documented contact to (suspected) SARS-CoV-2 positive patients during the relevant timeframe* (yes vs. no)	yes	28 (3.81–205.79)	0.001
Documented contact to (suspected) SARS-CoV-2 positive staff during the relevant timeframe* (yes vs. no)	yes	16 (2.12–120.65)	0.007

Nosocomial SARS-CoV-2 cases, where the first positive SARS-CoV-2 test was from a sample taken more than 10 days after hospital admission, were considered definite nosocomial cases. For SARS-CoV-2 cases, the relevant timeframe was defined as the 14 days preceding the first positive test. For controls, the relevant timeframe comprised of 14 days. Age represents the age in years at the beginning of the relevant timeframe. Contact patients were defined as patients placed in the same patient room for any period of time. Contact patient-days are the product of contact patients and the duration of contact in days. Abbreviations: CI95 – 95% confidence interval; ER – emergency room; ICU – intensive care unit; IQR – interquartile range. *Contact to more than one group of SARS-CoV-2 positive persons (e.g. patients and staff) was possible. In that case, both were recorded and counted

**Table 4:** Multivariable logistic regression analysis of definite nosocomial SARS-CoV-2 cases with matched controls (n = 76) and their respective controls (n = 76) for the outcome nosocomial SARS-CoV-2 acquisition

Parameter	Odds ratio (CI95)	*p *value
Present at a ward with a SARS-CoV-2 outbreak reported to authorities during the stay or up to 14 days later (yes vs. no)	15.89 (2.51–100.77)	0.003
Documented contact to a (suspected) SARS-CoV-2 case during the relevant timeframe (yes vs. no)	23.35 (4.63–117.72)	< 0.001

Nosocomial SARS-CoV-2 cases, where the first positive SARS-CoV-2 test was from a sample taken more than 10 days after hospital admission, were considered definite nosocomial cases

## Discussion

Our study revealed presence at a ward that experienced a SARS-CoV-2 outbreak as well as known contact to SARS-CoV-2 cases as the main risk factors for nosocomial SARS-CoV-2 infections in patients. Contact tracing to identify persons at high risk of developing SARS-CoV-2 due to prior exposition has been a key aspect of mitigation strategies since the beginning of the pandemic [30, 31]. Realities in hospitals, however, are sometimes complex and convoluted, and contact constellations are not always obvious. Fittingly, despite intensive contact tracing and investigation, for almost half of nosocomial patients, no prior contact to a positive person could be ascertained.

To be present at a ward that experiences a SARS-CoV-2 outbreak means being in an environment with a particularly high SARS-CoV-2 prevalence. The higher the prevalence, the higher the likelihood that besides the already identified cases, individuals infected but not yet identified are present, further contributing to the uncontrolled spread of the virus [32, 33]. We consider the fact that not only known contact to SARS-CoV-2 cases, but also the mere presence at an outbreak ward, significantly increased the risk of nosocomial SARS-CoV-2 infections, to be a clear indication to preemptively isolate not only patients that were knowingly exposed to cases, but also those treated at wards experiencing outbreaks. When transferring patients, it is imperative that this information is communicated to other wards or institutions. As demonstrated in various studies, transferred patients can introduce pathogens and be the source of outbreaks in receiving institutions [34–36]. To prevent this, it is crucial to systematically identify these patients and pass information on to others.

In a previous study, we highlighted the often underestimated infection risk originating from SARS-CoV-2 positive staff [37]. While, the results of the study at hand do not contradict our previous findings, they help to add nuance to preventive considerations, revealing that both groups, infected healthcare workers and infected patients, can lead to nosocomial SARS-CoV-2 cases and outbreaks. The large confidence intervals of the odds ratios determined by our multivariable analyses, which are attributable to the limited size of our study, illustrate that conclusions have to be made with caution. However, we consider our results and the above-stated interpretation to be in alignment with other available literature [38–40].

Since we intended to provide an all-encompassing overview of all potentially nosocomial cases in our hospital during the study period, we descriptively presented data from all patients that tested positive five days or later after admission. Approximately 8% of all SARS-CoV-2 patients treated during the study period were potentially nosocomial cases. This value is comparable to findings from previous studies [41, 42], and illustrates a considerable potential for reducing the burden of SARS-CoV-2 on healthcare by preventing nosocomial infections. It is important to recognize, however, that these represent a “high-end estimate” of patients who acquired SARS-CoV-2 during their hospitalization at our hospital. Given the median incubation period of four to five days [28, 29], it can be presumed that a substantial portion of patients counted as nosocomial, acquired the virus before being admitted to the hospital. To not confound deeper analyses with potentially community-acquired cases, we opted to limit the case–control study to cases that we considered definite nosocomial cases. We expected that patients with a higher number of movements (i.e. patient room or ward transfers) as well as documented procedures during the relevant timeframe would be at a significantly higher risk of nosocomial SARS-CoV-2 infection, particularly since other studies have identified patients in shared patient rooms to be at a higher risk for nosocomial SARS-CoV-2 infection [43]. To our surprise, the multivariable logistic regression analysis did not reveal these variables as risk factors. This allows for the cautious interpretation that patients do not have to be confined to their rooms and medical care does not be stopped, in order to prevent the spread of the virus. Patient treatment, if necessary involving transfers to other parts of the hospital, can continue and does not per se lead to nosocomial SARS-CoV-2 cases.

Various limitations have to be acknowledged when interpreting the study results. Data were collected retrospectively, which entails that certain pieces of information were no longer available at the time of data collection or might have been interpreted differently in a prospective setting. This is particularly relevant as data available for nosocomial cases were substantially more comprehensive than for controls. As mentioned above, nosocomial cases were extensively investigated at the time of diagnosis and the results of this research were documented systematically. Such systematic documentation was not available for controls. However, with regards to the main risk factors determined by our analyses, exposition to SARS-CoV-2 cases or outbreaks, we are confident that this information is accurately represented for controls as well, since all patients with known exposition to SARS-CoV-2 cases or clusters were systematically marked, even if they ultimately remained SARS-CoV-2 negative. When nosocomial cases and particularly clusters of cases were detected, extensive screening to identify further cases was performed. This also led to the detection of asymptomatically infected patients. Conversely, it is conceivable that asymptomatically infected patients without known association to other cases or outbreaks might have been under detected. Moreover, the incidence in the community during the study period was not considered, but has to be presumed to have influenced the occurrence of both community- and healthcare-associated SARS-CoV-2 infections. Another aspect that was not systematically collected and therefore not accounted for in our analyses, was the degree of adherence by healthcare workers to recommendations regarding personal protective equipment and hand hygiene, which both play an important role in preventing the spread of SARS-CoV-2. Lastly, vaccines were not yet widely available when the study was performed. Therefore, their mitigating effect on the spread of SARS-CoV-2 was not accounted for.

## Conclusions

Known contact to SARS-CoV-2 cases and association to SARS-CoV-2 outbreaks at the ward level were demonstrated to be the primary risk factors for nosocomial SARS-CoV-2 infections in patients. This highlights the importance of quickly identifying these patients and taking adequate preventive measures. Besides preemptive isolation of such patients from others, a consistent method to label high-risk patients in order to prevent loss of information should be implemented. Provided that such a system is in place, our findings suggest that routine patient care does not have to be paused in order to mitigate the spread of SARS-CoV-2 in hospitals.

## Data Availability

Not available, because all data were obtained in accordance with the German Protection Against Infection Act.

## Abbreviations

CI95: 95% Confidence interval
HAI: Healthcare-associated infection
IPC: Infection prevention and control
LRTI: Lower respiratory tract infection
OR: Odds ratio
RT-PCR: Reverse transcriptase polymerase chain reaction
